# The Effects of Fasting or Ketogenic Diet on Endurance Exercise Performance and Metabolism in Female Mice

**DOI:** 10.3390/metabo11060397

**Published:** 2021-06-18

**Authors:** Lola E. Holcomb, Caitlin C. O’Neill, Elizabeth A. DeWitt, Stephen C. Kolwicz

**Affiliations:** Heart and Muscle Metabolism Laboratory, Department of Health and Exercise Physiology, Ursinus College, Collegeville, PA 19426, USA; loholcomb@ursinus.edu (L.E.H.); caoneill@ursinus.edu (C.C.O.); lizzydewitt@gmail.com (E.A.D.)

**Keywords:** nutritional ketosis, lipid metabolism, ketone body metabolism, glycogen, triglycerides

## Abstract

The promotion of ketone body (KB) metabolism via ketosis has been suggested as a strategy to increase exercise performance. However, studies in humans and animals have yielded inconsistent results. The purpose of the current study was to examine the effects of ketosis, achieved via fasting or a short-term ketogenic diet (KD), on endurance exercise performance in female mice. After 8 h of fasting, serum KB significantly increased and serum glucose significantly decreased in fasted compared to fed mice. When subjected to an endurance exercise capacity (EEC) test on a motorized treadmill, both fed and fasted mice showed similar EEC performance. A 5-week KD (90% calories from fat) significantly increased serum KB but did not increase EEC times compared to chow-fed mice. KD mice gained significantly more weight than chow-fed mice and had greater adipose tissue mass. Biochemical tissue analysis showed that KD led to significant increases in triglyceride content in the heart and liver and significant decreases in glycogen content in the muscle and liver. Furthermore, KD downregulated genes involved in glucose and KB oxidation and upregulated genes involved in lipid metabolism in the heart. These findings suggest that a short-term KD is not an effective strategy to enhance exercise performance and may lead to increased adiposity, abnormal endogenous tissue storage, and cardiometabolic remodeling.

## 1. Introduction

Ketosis is a physiological metabolic state of elevated serum ketone bodies that may occur in situations of fasting/starvation [1], exercise [2], or diabetes [3,4]. In these situations, the serum ketone body concentrations in both humans and rodents rise from ~0.2 mM to above 3.0 mM and can rise to even higher levels in the pathological condition of ketoacidosis [5]. Although the use of ketone bodies for ATP production is generally low in the fed state, ketone bodies provide an alternative fuel source for the brain, heart, and muscle, particularly during nutrient deprivation and low carbohydrate availability. Moreover, recent works have identified that ketone body oxidation is upregulated in the hypertrophied and failing heart [6,7,8], which might serve as an alternative fuel source to sustain myocardial function.

The ketone bodies acetoacetate (AcAc) and β-hydroxybutyrate (β-OHB) are formed in liver mitochondria during ketogenesis, which requires acetyl-CoA, generated via the beta-oxidation of fatty acids. The ketogenic process continues with the aid of several enzymes, including mitochondrial acetyl-CoA acetyltransferase 1 (*Acat1*), 3-hydroxy-3-methylglutaryl-CoA synthase (*Hmgcs1*), Hmgc-CoA lyase, and mitochondrial beta-hydroxybutyrate dehydrogenase (*Bdh1*). AcAc and β-OHB are then released by the liver into the systemic circulation. Once inside the peripheral cell, β-OHB (the most highly concentrated ketone body) is rapidly oxidized via the enzymes *Bdh1*, succinyl-CoA:3-oxoacid-CoA transferase (Scot, encoded by *Oxct1*), and *Acat1* into acetyl CoA for subsequent entry into the tricarboxylic acid (TCA) cycle, ultimately providing reducing equivalents for the electron transport chain.

The utilization of ketone bodies as an energy source may be advantageous for exercising muscle as ketone bodies are suggested to be more energy-efficient than glucose or fatty acids [9,10]. In a physiological context, ketosis can be achieved by fasting, via exogenous supplementation or the consumption of a ketogenic diet (KD). Fasting studies in humans and animals generally restrict food from 12–16 h up to 48 h, which can increase serum ketone body concentrations but may elicit inconsistent effects on performance [11]. Exogenous ingestion of ketone bodies is an alternative method to rapidly achieve ketosis but may be an expensive option due to the purchase of supplements [12]. KDs typically supply approximately 80–90% of calories from fat, 10–15% calories from protein, and <5% calories from carbohydrates [10]. In theory, the high fat intake, combined with the low CHO intake, is purported to stimulate fat oxidation and promote fat loss, leading to its popularity for the treatment of obesity [13]. KDs have been used successfully in the clinic for the treatment of epilepsy and other neurological conditions since the 1920s [14]. Recently, increased interest from the scientific community suggests that KD may be effective in extending the lifespan of mice [15], as well as in weight loss in mice [1,16] and humans [17,18]. However, studies examining the effect of KD on liver steatosis [19,20], glucose homeostasis [19,20,21], and dyslipidemia [22,23] remain controversial.

Recent studies suggest that supplementation with ketone body esters improves exercise performance [24,25], anointing ketone bodies as a potential “super fuel”. These observations have extended research agendas to examine the effects of the KD as a strategy to improve exercise performance, which are summarized in a recent review [5]. Overall, much of the literature does not support the KD as a dietary strategy to enhance exercise performance. In addition, there are significant concerns within the scientific and medical communities regarding the potential negative consequences of consuming a diet that is substantially high in fats and extremely low in carbohydrates. However, much of the existing research has been conducted in a predominantly male population. Therefore, additional studies in females are required to examine the potential systemic and metabolic consequences of the KD.

Currently, limited studies focusing on females exist in the literature. After 4 weeks of a KD, female mice displayed higher fat-to-lean mass ratios and decreased cardiac function compared to chow-fed controls [26]. In human studies, females fed with a 4 week KD showed decreased time to exhaustion during a cycling test in a cross-over design [27], whereas 4 weeks of a KD led to decreased exercise performance in females but not in males [28,29]. These results suggest that females may be more adversely affected by changes in ketone body metabolism than males. Therefore, the purpose of this study was to investigate the effects of physiological ketosis, induced by short-term fasting, or nutritional ketosis, achieved through the short-term administration of a KD, on endurance exercise capacity and the metabolic phenotype in female mice.

## 2. Results

### 2.1. Fasting Induces Physiological Ketosis but Does Not Improve Endurance Exercise Capacity

**Figure 1:**
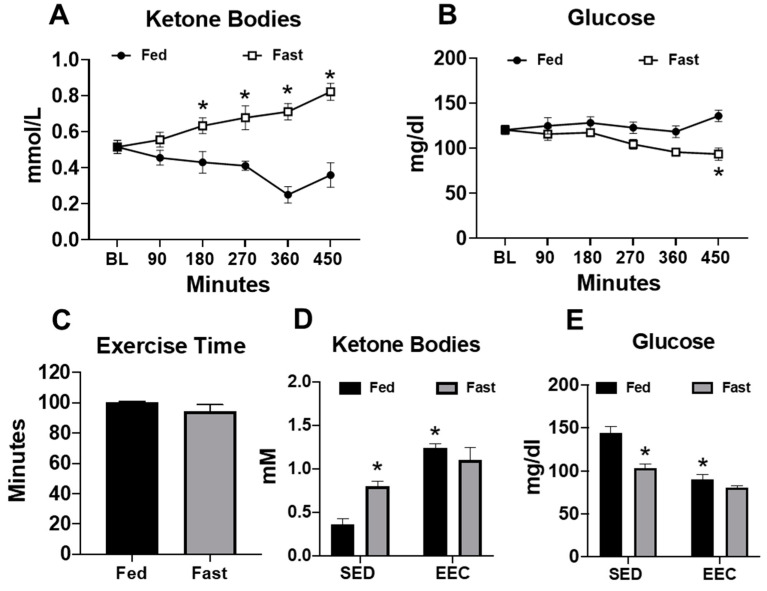
Serum ketone bodies increase in response to fasting and/or endurance exercise. (**A**) Serum ketone bodies and (**B**) serum glucose levels measured in fed and fasted mice (*n* = 9–10 per group); (**C**) exercise time in the endurance exercise capacity (EEC) test in fed and fasted mice (*n* = 5 per group); (**D**) serum ketone bodies; and (**E**) serum glucose in fed and fasted mice at the end of the exercise capacity test (*n* = 5 per group). Non-exercised, sedentary (SED) fed and fasted mice used for comparison (*n* = 7 per group). * *p* < 0.05 vs. fed or fed-SED.

Female mice were subjected to a short-term fast for approximately 8 h in order to induce physiological ketosis. After 180 min of fasting, serum ketone body levels began to increase significantly and remained near 0.8 mM at the end of the fasting period (Figure 1A). At the end of the fasting period, mice experienced a significant decrease in blood glucose levels (Figure 1B). These data demonstrate that fasting of up to 8 h is sufficient to induce physiological ketosis. To determine whether physiological ketosis improved exercise performance, we subjected mice to an endurance exercise capacity test on a motorized treadmill. Despite the presence of elevated ketone bodies prior to exercise, fasted mice achieved similar exercise times as their fed counterparts (Figure 1C). Interestingly, endurance exercise significantly elevated the serum ketone bodies in fed mice, whereas blood glucose levels were lowered to a similar level as those of fasted mice (Figure 1D,E). These findings suggest that physiological ketosis, induced by a short-term fast, is insufficient to improve endurance exercise performance, possibly because endurance exercise promotes ketosis independently of fasting.

### 2.2. Fasting Does Not Alter Endogenous Triacylglycerol or Glycogen Content in the Heart, Gastrocnemius, or Liver

**Figure 2:**
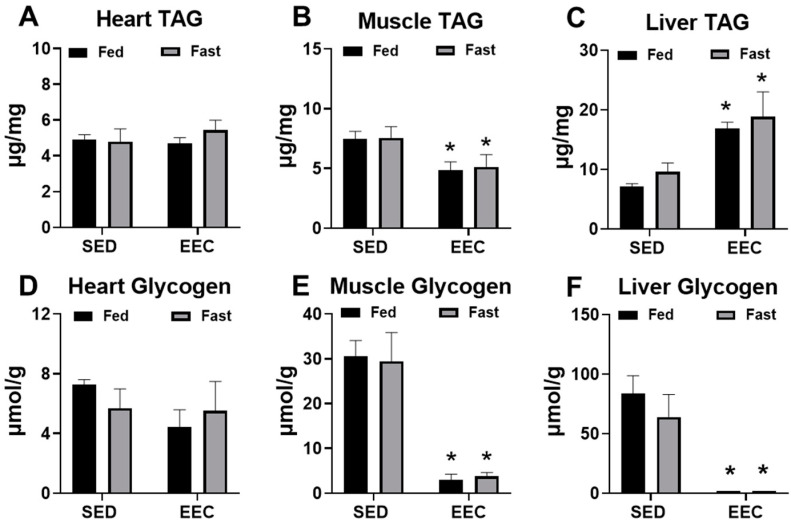
Fasting does not significantly alter triacylglycerol or glycogen stores in tissue. (**A**) Heart, (**B**) gastrocnemius, and (**C**) liver triacylglycerol (TAG) content in fed and fasted female mice. Tissues were harvested immediately following an endurance exercise capacity (EEC) test (*n* = 3–4 each group). (**D**) Heart, (**E**) gastrocnemius, and (**F**) liver glycogen content in fed and fasted female tissues harvested immediately following the EEC (*n* = 3–4 each group). A cohort of sedentary, non-exercised (SED) fasted and fed mice were used for comparison (*n*= 4–6 each group). * *p* < 0.05 vs. SED.

At the end of the EEC, heart, gastrocnemius, and liver tissue was harvested from fed and fasted mice to determine changes in the endogenous storage of triacylglycerol (TAG) and glycogen after exhaustive exercise. As shown in Figure 2, short-term fasting did not significantly alter TAG or glycogen content in the heart, gastrocnemius, or liver tissue. At the conclusion of exercise, TAG content in the gastrocnemius was significantly decreased (Figure 2B) and in the liver was significantly increased (Figure 2C) to a similar extent in fed and fasted mice. Exhaustive exercise also led to significant depletions of muscle and liver glycogen with no significant differences between the fed and fasted state (Figure 2E,F). Overall, these findings suggest that short-term fasting of approximately 8 h does not alter the response of tissue glycogen or TAG content to endurance exercise.

### 2.3. Short-Term Ketogenic Diet Increases Weight Gain and Adiposity

**Figure 3:**
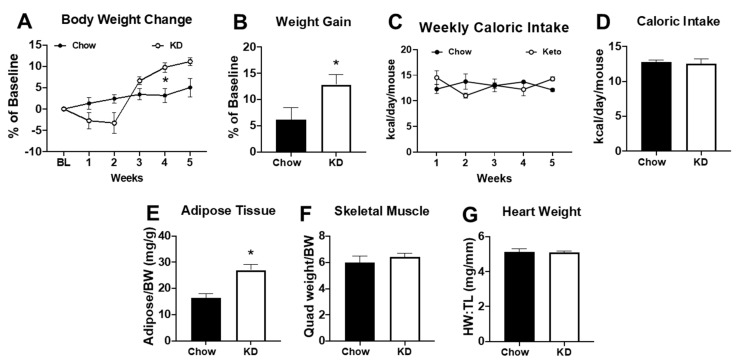
Five weeks of a ketogenic diet increased body weight and adiposity. (**A**) Body weight change relative to baseline, (**B**) percentage of weight gain relative to baseline, (**C**) weekly caloric intake, and (**D**) average caloric intake over 5 weeks in chow-fed (*n* = 6) and ketogenic diet (KD, *n* = 9) mice; (**E**) adipose tissue weight (from the inguinal fat pad) normalized to body weight (BW), (**F**) skeletal muscle weight (from the quadriceps) normalized to body weight (BW), and (**G**) heart weight normalized to tibia length (HW:TL) in chow (*n* = 6) or ketogenic diet (*n* = 9). * *p* < 0.05 vs. chow.

Since acute changes in serum ketone bodies were unable to increase endurance exercise performance, we evaluated whether administration of a short-term (i.e., 5 weeks) ketogenic diet (KD) was a more effective strategy. After 2 weeks of the KD, mice experienced a weight loss of ~5% (Figure 2A, *p* = NS). Of note, three out of 12 mice (25%) experienced a weight loss of >20% with significantly decreased tissue weights and were removed from the study (data not shown). During the last 3 weeks of the KD, the remaining mice regained their body weight and, by the end of the study, had a significantly greater body weight relative to baseline values (Figure 3A,B). Caloric intake in KD mice was reduced during the second week of the diet (Figure 3C) but did not lead to a significant difference in average caloric intake over the 5-week period (Figure 3D). In addition to having a greater body weight, KD mice had a higher adipose tissue mass compared to chow-fed controls (Figure 3E) with no significant differences in skeletal muscle weight or heart weight (Figure 3F,G). These data show that KD causes an initial transient weight loss but may result in increased adiposity as the diet continues.

### 2.4. Short-Term Ketogenic Diet Elevates Serum Ketone Bodies and Total Cholesterol

**Figure 4:**
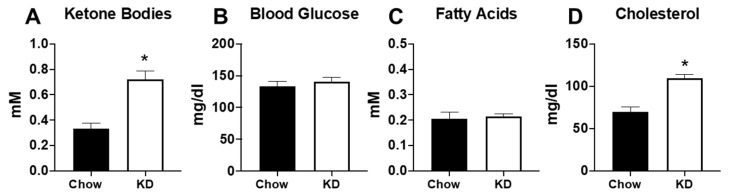
Five weeks of the ketogenic diet increased serum ketone bodies and cholesterol. (**A**) Serum ketone bodies; (**B**) serum glucose; (**C**) serum fatty acids, and (**D**) total cholesterol measured in chow-fed (*n* = 6) and ketogenic diet (KD, *n* = 9) mice. * *p* < 0.05 vs. chow.

As expected, 5 weeks of KD led to significantly elevated serum ketone body levels (Figure 4A). Although no significant differences were noted in blood glucose or serum fatty acids (Figure 4B,C), 5 weeks of KD led to significant increases in total cholesterol compared to the chow-fed mice (Figure 4D). These findings support previous studies demonstrating elevations in serum cholesterol following administration of a KD [16,23].

### 2.5. Short-Term Ketogenic Diet Depletes Glycogen and Increases Tissue Triglycerides

**Figure 5:**
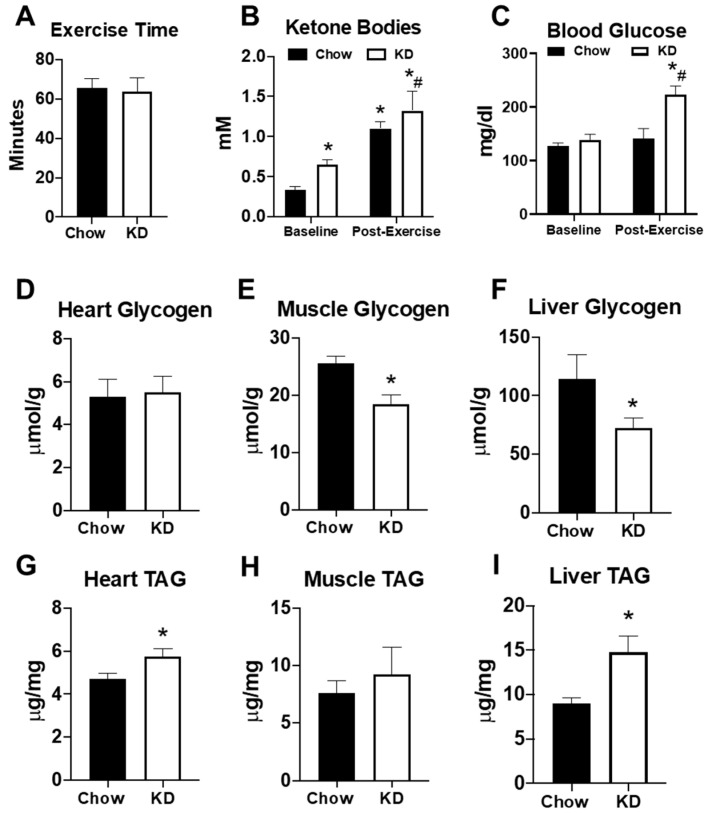
The effects of a ketogenic diet on exercise and endogenous metabolism. (**A**) Exercise time in the endurance exercise capacity test in chow-fed (*n* = 6) and ketogenic diet (KD, *n* = 4) mice; (**B**) serum ketone bodies and (**C**) serum glucose levels in chow (*n* = 6) and ketogenic diet (*n* = 4) mice measured at the beginning (baseline) and at end of the exercise capacity test (post-exercise). Glycogen content measured in the (**D**) heart, (**E**) gastrocnemius, and (**F**) liver in chow-fed (*n* = 6) and ketogenic diet (*n* = 9) mice; triacylglycerol (TAG) content measured in the (**G**) heart, (**H**) gastrocnemius, and (**I**) liver in chow-fed (*n* = 6) and ketogenic diet (*n* = 9) mice. * *p* < 0.05 vs. chow or baseline. # *p* < 0.05 vs. chow-post-exercise.

To determine the impact of the KD on exercise performance, mice were subjected to an endurance exercise capacity test on a motorized treadmill. As observed with fasting, endurance exercise times were similar in both chow-fed and KD mice (Figure 5A). Endurance exercise increased serum ketone bodies in both chow-fed and KD mice (Figure 5B). However, both serum ketone bodies and blood glucose were significantly higher in KD mice at the end of exercise (Figure 5B,C). To further examine the impact of the KD on endogenous metabolism, we analyzed glycogen and triacylglycerol (TAG) content in the heart, gastrocnemius, and liver approximately 72 h after the endurance exercise test. Although heart glycogen was similar, the KD led to a significant depletion of glycogen in the gastrocnemius and liver (Figure 5D–F). KD mice also had significantly elevated TAG in the heart and liver, but not the gastrocnemius (Figure 5G–I). These data show that a short-term KD does not increase endurance exercise performance, despite elevations in serum ketone bodies. Moreover, the data suggest that a short-term KD may lead to glycogen depletion and lipid accumulation in some organs.

### 2.6. Short-Term Ketogenic Diet Alters Gene Expression in the Heart, Skeletal Muscle, and Liver

**Figure 6:**
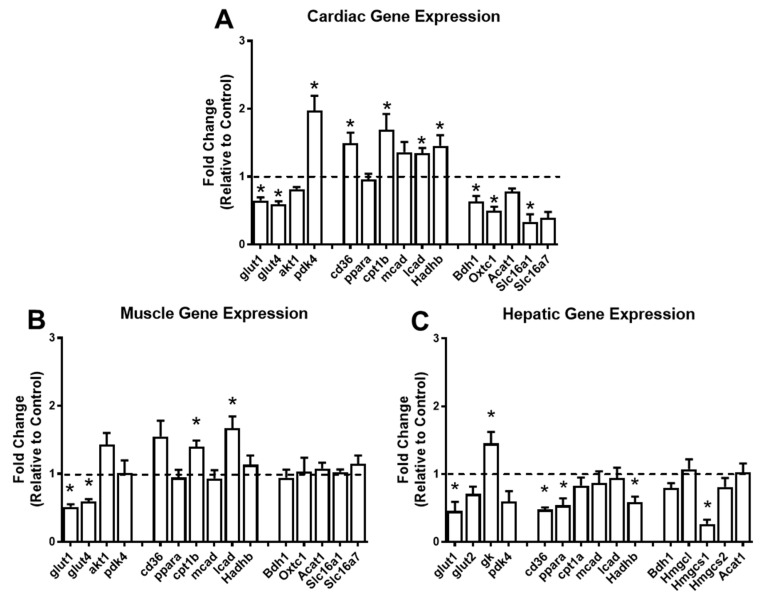
The ketogenic diet alters the expression of cardiac, skeletal muscle, and hepatic genes. Gene expression analysis conducted via RT-PCR in the (**A**) heart (*n* = 5 per group); (**B**) gastrocnemius (*n* = 4 per group); and (**C**) liver (*n* = 4 per group). For each tissue, genes involved in glucose, lipid, and ketone body metabolism were analyzed. Glucose metabolism: *glut1*, glucose-transporter 1; *glut2*, glucose-transporter 2; *glut4*, glucose-transporter 4; *akt1*, AKT serine/threonine kinase 1; *gk*, glucokinase; *pdk4*, pyruvate dehydrogenase kinase 4. Fatty acid metabolism: *cd36*, cluster of differentiation 36; *ppara*, peroxisome proliferator-activated receptor alpha; *cpt1b*, carnitine palmitoyltransferase 1B; *cpt1a*, carnitine palmitoyltransferase 1A; *mcad*, medium chain acyl-CoA dehydrogenase; *lcad*, long-chain acyl-CoA dehydrogenase; *hadhb*, hydroxyacyl-CoA dehydrogenase trifunctional multienzyme complex subunit beta. Ketone body metabolism: *bdh1*, 3-hydroxybutyrate dehydrogenase 1; *Oxct1*, 3-oxoacid CoA-transferase 1; *Acat1*, acetyl-CoA acetyltransferase 1; *Slc16a1*, monocarboxylate transporter 1; *Slc16a7*, monocarboxylate transporter 2; *Hmgcl*, 3-hydroxy-3-methylglutaryl-CoA lyase; *Hmgcs1*, hydroxymethylglutaryl-CoA synthase 1; and *Hmgcs2*, hydroxymethylglutaryl-CoA synthase. * *p* < 0.05 vs. chow.

To understand the impact of a short-term KD on the pathways of glucose, lipid, and ketone body metabolism, we performed gene expression analysis in the heart, gastrocnemius, and liver. In the heart, the KD significantly downregulated the expression of the glucose transporters *Glut1* and *Glut4*, and upregulated the expression of *Pdk4*, which is a kinase that inactivates pyruvate dehydrogenase, and subsequently inhibits glucose oxidation (Figure 6A). Concomitantly, genes involved in myocardial fatty acid uptake within the myocyte (*Cd36*) and mitochondria (*Cpt1β*), as well as genes involved in beta-oxidation (*Lcad* and *Hadhb*), were significantly upregulated (Figure 6A). Furthermore, *Bdh1*, *Oxct1*, and *Slc116a1*, genes involved with ketone body oxidation, were significantly downregulated in the heart in response to the KD (Figure 6A). In the skeletal muscle, the KD led to significant decreases in *Glut1* and *Glut4* and significant increases in *Cpt1β* and *Lcad* (Figure 6B). None of the genes involved with ketone body oxidation were altered (Figure 6B). In the liver, *Glut1* was downregulated, and glucokinase (*Gk*), a glycolytic gene, was upregulated in response to the KD (Figure 6C). The lipid metabolism genes *Cd36*, *Pparα*, and *Hadhb*, and the ketogenic gene *Hmgcs1* were downregulated in the liver in response to the KD (Figure 6C). Taken together, these findings suggest that a short-term KD has profound effects on the expression of genes involved in cardiac glucose, lipid, and ketone body metabolism, with relatively minor changes in the skeletal muscle and liver.

## 3. Discussion

There are several major findings from the present study. First, physiological ketosis, induced by short-term fasting (~8 h) or nutritional ketosis, induced by short-term KD (5 weeks), are ineffective in improving endurance exercise performance in female mice. Second, the KD leads to a transient weight loss in the early course of the intervention. However, as the diet continues, weight gain occurs and ultimately leads to an increase in adiposity. Third, the KD may lead to a variety of untoward side effects, such as hypercholesterolemia, lipid accumulation in the heart and liver, and glycogen depletion in the skeletal muscle and liver. Lastly, the KD may result in metabolic remodeling in the heart by downregulating genes involved in glucose and ketone body oxidative pathways and upregulating lipid utilization pathways. Overall, these findings would advise against the KD as a strategy for the enhancement of weight loss or endurance exercise performance, especially in females.

We previously published a review article on the effects of KD on exercise performance [5]. Our review of the literature generally found that the KD was not an effective strategy to improve aerobic [30,31,32,33] or anaerobic [34,35,36,37] performance in human subjects. However, studies in rodent models were mixed, with observations of enhancements [38,39,40], impairments [26], and no change [41,42,43] in exercise performance. Studies in humans present certain limitations, such as controlling dietary intake and obtaining tissue for investigation and molecular insights. Since dietary intervention can be well controlled in animals, additional studies, particularly in female populations, are warranted to provide definitive conclusions. Consistent with studies in humans [30,31,32,33] and male rodents [41,42,43], we found no benefit of a short-term KD on endurance exercise performance. Several key data may explain this observation. First, female mice fed with the KD experienced a greater increase in body weight and adipose tissue mass, which has been previously reported [26]. Although this overweight/obese phenotype did not impair performance, this would certainly not provide an advantage. Second, the KD was associated with an upregulation of genes involved in lipid metabolism but not ketone body oxidation in the skeletal muscle. Although not directly assessed in this study, this may reflect a lack of increased ketone body oxidation in the exercising skeletal muscle. Third, mice fed with a KD had lower glycogen levels in the muscles and liver. Since muscle and liver glycogen are important glucose sources during exercise, these presentations are likely to be limiting factors for endurance exercise [44]. Therefore, our findings demonstrating the inability of the KD to enhance endurance exercise are not surprising. Of note, we observed a hyperglycemic response to exercise in the female mice fed with a KD. Whether this response is due to decreased cardiac and skeletal muscle uptake of glucose or impaired insulin signaling is not clear, but glucose intolerance in mice fed with the KD for 6 weeks and decreased insulin sensitivity in rats fed with the KD for 8 weeks has been reported [20,45].

Recent studies suggest that an increased reliance on ketone body metabolism via exogenous supplementation may be effective in improving endurance exercise performance [24], although these findings are equivocal [46,47,48]. Fasting has been suggested as a method to increase endurance exercise performance via a potential glycogen-sparing effect and an increase in substrate availability in the form of fatty acids and the ketone body beta-hydroxybutyrate [49,50]. Since fasting times of greater than 24 h generally have a negative effect on exercise performance [51], we elected to explore the effect of short-term fasting on ketosis and endurance exercise performance. Our data show a gradual increase in the serum ketone body concentration during fasting, with a peak of ~0.8 mM after approximately 8 h. However, this level of ketosis is insufficient to improve endurance exercise time. There are a few observations that may explain our finding. First, we measured ketone bodies at the end of the endurance exercise capacity test and found that both fed and fasted mice had similar serum concentrations. This “exercise-induced ketosis” is likely due to increased lipolysis and a compensatory hepatic ketone body synthesis. Since the natural response to exercise is an elevation of serum ketone bodies, it would make sense that starting the exercise session in ketosis may not provide an advantage, particularly as glucose is an integral source of energy during the early course of exercise [52]. These findings argue against the effectiveness of ketone body supplements. However, an important distinction should be made as our ketone body levels were only in the 1.0–1.5 mM range, whereas previous studies using ketone body supplements report significantly higher serum levels [24,46,47]. Therefore, higher serum ketone body levels may be required to distinguish endurance exercise effects.

During the course of our studies, we identified several side effects of the KD that are of potential concern. First, although an initial weight loss occurred in the early course of the diet, a rebound weight gain and increased adiposity were observed. The translational impact of this finding to humans is not clear. However, since the KD is a high-fat diet, consumption of high-calorie food sources, particularly in the face of a sedentary lifestyle, could present an unexpected consequence. As such, future human studies should also consider performing body composition assessments. Second, our observations of increased serum cholesterol and lipid accumulation in the heart and liver are of concern. Previous studies have identified elevations in serum lipids, particularly LDL cholesterol, in both humans and animals [22,23,26,53]. Although our data are limited to total cholesterol levels, an increase in the cholesterol subtypes would be expected due to the high dietary fat intake of the diet. An increased dietary fat intake has been associated with increased triacylglycerol levels in the heart and liver in mice [54]. Since lipid accumulation in the heart and liver is associated with cardiolipotoxicity [55] and liver steatosis [56], respectively, the consumption of the KD may represent a deleterious strategy. However, the KD is suggested to be an effective intervention for nonalcoholic fatty liver disease (NAFLD) [57]. The role of the KD in models of cardiolipotoxicity is not known, so additional research in this area may be necessary.

Over the last several years, a number of studies have focused on cardiac ketone body metabolism, particularly in the setting of heart failure [6,8,58]. Overall, these studies demonstrate that ketone body oxidation is a significant substrate for ATP synthesis, which appears to increase in the setting of cardiac hypertrophy and heart failure [6,58]. Furthermore, enhancing ketone body metabolism via KD or beta-hydroxybutyrate infusion appears to be beneficial for cardiac remodeling and function [8]. Although we did not directly assess cardiac function and substrate metabolism in the present study, we evaluated the expression of several genes involved in glucose, lipid, and ketone body metabolism in heart, gastrocnemius, and liver tissue in female mice subjected to 5 weeks of a KD. Overall, our findings suggest that the KD has profound effects on cardiac metabolic pathways by downregulating genes involved in glucose and ketone body oxidation and upregulating genes involved in fatty acid uptake and beta-oxidation. Since hypertrophied and failing hearts are known to downregulate lipid metabolism, while concomitantly increasing glucose and ketone body metabolism [59,60,61], it is possible that the KD does not act by enhancing ketone body oxidation, but rather by normalizing the impaired lipid metabolism. Further studies evaluating the cardiometabolic consequences of the KD in the setting of cardiac hypertrophy and failure should help to elucidate this hypothesis.

There are design considerations in the present study that should be noted. First, we performed this study on female mice specifically due to their under-representation in animal research and the limited availability of female KD studies. One reason females may be used less frequently in research is the potential for estrogen and other sex hormones to influence outcomes and increase variability [62]. Although we did not account for sex hormones in our study, reports suggest that the menstrual cycle has minimal effects on performance in female athletes [63,64] and the estrus cycle has subtle effects on exercise performance in female mice [65]. Second, the fatty acid composition of the KD used in the present study consisted predominantly of unsaturated fatty acids, with an abundance of polyunsaturated fatty acids. There is a possibility that a KD consisting of differing dietary fatty acid content and composition could influence the overall findings. For example, the fatty acid composition was an important factor in the development of diastolic dysfunction in mouse models of diet-induced obesity [66]. Additional studies will help to elucidate these potential differences.

In summary, in the current study we evaluated the effects of physiological ketosis, induced by short-term fasting, or nutritional ketosis, caused by 5 weeks of a KD, on endurance exercise performance in female mice. Our data show that neither strategy is capable of prolonging endurance exercise. We also report several potential negative side effects of the KD, including increased adiposity, hypercholesterolemia, glycogen depletion in the skeletal muscle and liver, and lipid accumulation in the heart and liver. In addition, we observed exercise-induced hyperglycemia in KD-fed females, which may suggest potential abnormal glucose homeostasis. Our study also evaluated the consequences of short-term KD on the expression of genes involved in glucose, lipid, and ketone body metabolism in the heart, gastrocnemius, and liver. We report significant changes in genes responsible for metabolic pathways in the heart, particularly a downregulation of glucose and ketone body utilization and an upregulation of lipid metabolism. Overall, our study does not support the state of ketosis for the enhancement of endurance exercise and warns against the use of the KD through the identification of several potential negative side effects.

## 4. Materials and Methods

### 4.1. Animals

All experimental protocols used in this study were approved by the Institutional Animal Care and Use Committee of Ursinus College (Animal Welfare Assurance: #D16-00678; IACUC Protocol: 81 #2017-ModA-SK). Female, C57BL6-NCrl mice were obtained from an in-house animal colony. Mice in the fed and fasting experiments were 12–14 weeks of age. For this study, food was removed at 0900 h during the light cycle. Blood glucose and ketone bodies were measured at baseline and at 90-min intervals after food restriction. In a separate cohort, mice at 12–14 weeks were fed with a ketogenic diet (KD) or standard chow for 5 weeks. Body weight was measured weekly. Mice were housed 3–5 mice per cage and maintained on a 12-h light/dark cycle. Food and water were provided ad libitum except for the fasting protocol, in which food was removed for ~8 h during the light cycle.

### 4.2. Composition of Diet

The KD (Envigo Teklad Diets, TD.96355, Madison, WI, USA) consisted of 90.5% calories from fat, 0.3% calories from carbohydrates, and 9.2% calories from protein. The fatty acid composition consisted of 73% unsaturated (22% monounsaturated, 51% polyunsaturated) and 27% saturated fatty acids. The standard chow diet (LabDiet, #5001, St. Louis, MO, USA) consisted of 13.6% calories from fat, 57.9% calories from carbohydrates, and 28.7% calories from protein. Caloric intake was estimated based on the difference in the weight of the food provided and the weight of the food remaining after a two-day period. The weight of the food was converted to calories based on the manufacturer’s reported caloric yield: 3.36 kcal/g for chow and 6.7 kcal/g for KD.

### 4.3. Serum Analyses

Blood glucose was measured via a hand-held glucometer (Contour, Ascensia Diabetes Care, Parsippany, NJ, USA) using blood obtained from the tail tip. Serum ketone bodies were measured using a hand-held meter (Keto-Mojo, Keto-Check, Inc., Napa, CA, USA) from the tail tip. Serum fatty acids and cholesterol were measured in blood harvested from the chest cavity using commercially available kits (Wako Diagnostics, Mountain View, CA, USA) per the manufacturer’s instructions.

### 4.4. Endurance Exercise Capacity Tests

Mice from the fed vs. fasted and KD vs. chow cohorts were subjected to an endurance exercise capacity (EEC) test on a motorized treadmill adapted from a previous study [67]. Mice were monitored by blinded researchers and motivated to run via non-aversive stimuli [68]. The EEC protocol consisted of a graded protocol of increasing speeds, starting at 10 m/min for 15 min. The speed was increased to 12.6 m/min for an additional 15 min. Speeds were then increased by 2.4 m/min in 15-min intervals. The treadmill grade was constant at 10% throughout the protocol. The EEC was completed when the mice were unable to run continuously on the treadmill for at least 10 s or the final stage was completed. Prior to the EEC, all mice were acclimated on the treadmill for 4 consecutive days for 10 min at a speed of 10 m/min. All exercise sessions occurred between 1700 and 1900 h, near or at the start of the dark cycle. At the immediate conclusion of the EEC, fed and fasted mice were anesthetized with sodium pentobarbital (170 mg/kg). Serum was collected and tissues (heart, epididymal adipose tissue, quadriceps, and gastrocnemius) were removed and frozen in liquid nitrogen. All samples were stored at −80 °C. Chow-fed and KD mice had blood glucose and ketone body concentrations assessed at the beginning and end of the EEC test. At the conclusion of the EEC test, mice were allowed to recover in the animal facility for approximately 72 h, prior to further analysis.

### 4.5. Gravimetric Analysis

After the EEC, KD and chow fed mice were returned to the animal facility for approximately 72 h. After the recovery period, mice were injected with sodium pentobarbital (170 mg/kg, IP). After confirming sedation with the toe-pinch test, serum was collected, and tissues (heart, inguinal fat pad, quadriceps, and gastrocnemius) were removed and weighed. For cardiac hypertrophy comparisons, the heart weight to tibia length (HW:TL) ratio was determined. For other size comparisons, the quadriceps weight or adipose tissue to body weight ratio was determined. After weighing, tissues were frozen in liquid nitrogen and stored at −80 °C.

### 4.6. Biochemical Analysis in Tissues

Glycogen content was assessed in frozen heart, gastrocnemius, and liver tissues by determining the amount of glucose released from glycogen with a commercially available kit (Sigma Aldrich, GAHK-20, St. Louis, MO, USA). Glycogen was separated from exogenous glucose in the tissue using an alkaline extraction procedure [69]. Triglycerides were extracted and measured from frozen heart, gastrocnemius, and liver tissue using a triglyceride colorimetric assay kit (Cayman Chemicals, #10010303, Ann Arbor, MI, USA).

### 4.7. Analysis of Gene Expression

RNA was extracted from heart, gastrocnemius, and liver tissues using the RNeasy Mini Kit (Qiagen, #74104, Hilden, Germany). Complementary DNA (cDNA) synthesis was performed using omniscript reverse transcriptase and random hexamers, according to manufacturer’s instructions (Omniscript RT Kit, Qiagen, Hilden, Germany). Real-time polymerase chain reaction (RT-PCR) analysis was performed using SYBR-Green (Bio-Rad, Hercules, CA, USA). The mRNA levels of genes involved in glucose, lipid, and ketone body metabolism were normalized to 18s rRNA. Primer sequences have been previously published [70]. Values were determined as a fold change over controls.

### 4.8. Statistical Analysis

All data are presented as means ± standard error (SEM). Statistical analysis was tested using a Student’s *t*-test for all two group comparisons. For 4-group comparisons, a two-way ANOVA with Bonferroni post-hoc analysis was used. For longitudinal data, a two-way repeated measures ANOVA with a Bonferroni post-hoc test was used. Statistical analysis was tested at the *p* < 0.05 level. All graphs and analyses were performed using GraphPad Prism 8.0.

## Data Availability

The authors declare that the data supporting the findings of the study are available within the article.
